# HIV-1 Tat and gp120 as key drivers of neurodegeneration in the central nervous system

**DOI:** 10.3389/fmicb.2026.1895162

**Published:** 2026-08-12

**Authors:** Riccardo Cecchetto, Erica Diani, Virginia Lotti, Asia Palmisano, Anna Lagni, Marco Mantoan, Stefania Turrina, Dario Raniero, Giovanna Paolone, Annarita Mazzariol, Davide Gibellini

**Affiliations:** 1 Section of Microbiology, Department of Diagnostics and Public Health, University of Verona, Verona, Italy; 2 Section of Hygiene and Preventive, Environmental and Occupational Medicine, Department of Diagnostics and Public Health, University of Verona, Verona, Italy; 3 Section of Forensic Medicine, Department of Diagnostics and Public Health, University of Verona, Verona, Italy; 4 Section of Pharmacology, Department of Diagnostics and Public Health, University of Verona, Verona, Italy; 5 UOC Microbiology, AOUIVerona, Verona, Italy

**Keywords:** brain, CNS, gp120, hand, HIV, neurodegeneration, Parkinson’s disease, Tat

## Abstract

Combined antiretroviral therapy (cART) has deeply changed the approach to HIV disease treatment. cART tackles HIV replication and improves the life expectancy of HIV-infected people. Notwithstanding the effectiveness of cART in HIV infection control, several observations have determined that 15–50% of HIV-infected people display HIV-associated neurocognitive disorders (HAND) even under long-term viral suppression. Persistent production of the viral proteins Tat and gp120 by central nervous system reservoirs drives chronic neurotoxicity due to their remarkable extracellular stability and efficient uptake by neurons and glial cells. In this review, we will discuss current evidence on the molecular mechanisms by which extracellular Tat and gp120 orchestrate neurodegeneration. Four principal interconnected pathways emerge: (i) mitochondrial dysfunction; (ii) synaptodendritic injury; (iii) chronic neuroinflammation; and (iv) crosstalk with Alzheimer’s disease (AD) pathways. Converging data achieved from *in vitro* models, Tat and gp120-transgenic mice, post-mortem tissue, and cerebrospinal fluid (CSF) biomarkers indicate that these viral proteins contribute to frontostriatal atrophy and hybrid HAND-AD phenotypes increasingly observed in aging HIV patients. Understanding these mechanisms highlights the need for adjunctive neuroprotective strategies targeting CXCR4/CCR5 signaling, mitochondrial quality control, inflammatory pathways, and Aβ/Tau homeostasis derangement and supports the integration of multimodal neuroimaging and CSF proteomics in future longitudinal studies aimed at improving diagnosis and therapeutic development.

## 1 Introduction

The Human Immunodeficiency Virus (HIV) is a member of the *Retroviridae* family, classified as a lentivirus, due to its ability to cause persistent chronic infections (Masenga et al., 2023). HIV is the causative agent of Acquired Immune Deficiency Syndrome (AIDS), and its genome consists of two copies of single-stranded, positive-sense RNA approximately 9.7 kb in length, which encodes structural and non-structural proteins essential for viral replication. Two HIV genotypes were isolated and subsequently classified as HIV-1 and HIV-2. HIV-1 is the most widespread worldwide (about 95% of all HIV positive patients) and shows the highest clinical impact. While HIV-2 is endemic in Western Africa, it is less transmissible and exhibits a slower progression to AIDS than HIV-1. The primary targets of HIV are CD4-expressing cells, including CD4 + helper T cells, macrophages, and dendritic cells, which serve as primary infection reservoirs (Masenga et al., 2023). The cell infection process involves the binding of viral envelope glycoprotein gp120 to the CD4 receptor on CD4 + T-helper cells and macrophages, and chemokine coreceptors CXCR4 or CCR5, thereby determining viral entry (Kwong et al., 1998). The viral RNA genome is retrotranscribed into cDNA in the cytoplasm and then is integrated into the host cell genome, allowing for lifelong infection and evasion of host immune responses. The complexity of the replication cycle and the high mutation rates during retro-transcription by viral reverse transcriptase contribute to genetic diversity and immunological escape (Santoro and Perno, 2013).

According to the latest UNAIDS (Joint United Nations Programme on HIV/AIDS) update, approximately 40.8 million people worldwide are living with HIV, with an estimated 1.3 million new infections/year (last update on February 25, 2026) (UNAIDS, 2026).

The current first-line therapy used for HIV patients aims to control viral replication and is based on the use in combination of three drugs from at least two different antiretroviral drug classes, called combined antiretroviral therapy (cART). This treatment strategy has successfully counteracted the disease evolution and improved the life expectancy, transforming HIV-1 infection from a lethal to a chronic infection. Notwithstanding the direct derangement of the immune system, HIV-1 also alters different organs and tissues, including the cardiovascular system, hematopoietic lineages, bone, and central nervous system (CNS). In particular, HIV-infected patients can develop HIV-associated neurocognitive disorders (HAND), whose underlying pathogenic mechanisms show significant similarities to those implicated in Alzheimer’s and Parkinson’s diseases (Canet et al., 2018; Smith et al., 2019). HAND comprises a spectrum of neurological manifestations ranging from asymptomatic neurocognitive impairment to HIV-associated dementia, and is characterized by deficits in attention, executive functions, memory, and motor performance. The pathogenesis of HAND involves chronic neuroinflammation, disruption of brain homeostasis, glial activation, glutamate dysregulation and excitotoxicity, ultimately leading to neuronal and synaptic injury (Elbirt et al., 2015). Some studies reported that HAND persisted in 15–50% of HIV-infected individuals, with substantial variability across studies due to differences in diagnostic criteria, patient populations, access to antiretroviral treatment, and the increasing proportion of aging individuals living with HIV (Cysique and Brew, 2011; Elbirt et al., 2015; Zayyad and Spudich, 2015). Among the mechanisms implicated in HAND pathogenesis, the neurotoxicity related to HIV replication, viral proteins release, in particular Tat and gp120 are considered major drivers of neuronal dysfunction and neurodegeneration (Hellmuth et al., 2015). Host genetic factors, including specific HLA-C variants, may further influence susceptibility to neurocognitive impairment (Zipeto et al., 2018). In fact, HIV establishes long-lived reservoirs within the CNS, mainly in microglia and perivascular macrophages, contributing to chronic neuroinflammation through intermittent viral reactivation despite efficient viral suppression achieved by cART (Hellmuth et al., 2015; Sonti et al., 2021). The brain is considered one of the sanctuary reservoirs for HIV, due to its reduced immune surveillance and lower drug penetration (Sadowski and Hashemi, 2019), due to the presence of a natural anatomical barrier, the blood–brain barrier (BBB). HIV-1 enters the brain primarily through HIV-1-infected monocytes that pass across the BBB. These monocytes differentiate into macrophages, which harbor HIV-1 progeny and spread the virus in the CNS. Interestingly, the major permissive cellular targets of HIV-1 infection in the brain are represented by perivascular macrophages and microglia (Bagasra et al., 1996; Takahashi et al., 1996; Wiley, 1996; An et al., 1999; Wang et al., 2001). In addition, some studies have detected the virus in astrocytes, even though the entry mechanisms and the true role of these cells remain largely unclear (Valdebenito et al., 2021).

Within the CNS, microglia and perivascular macrophages represent the main immune cell populations and the principal targets of productive HIV infection, while astrocytes play essential roles in maintaining neuronal metabolism, synaptic function, and glutamate homeostasis (Zayyad and Spudich, 2015; Saylor et al., 2016). In contrast, neurons and oligodendrocytes are generally not considered major targets of HIV infection but are highly susceptible to indirect injury mediated by viral proteins and inflammatory responses. Chronic activation of microglia and astrocytes promotes the release of pro-inflammatory cytokines, reactive oxygen species, and excitotoxic mediators, leading to oxidative stress, neuronal dysfunction, oligodendrocyte damage, and white matter loss. These processes contribute to persistent neuroinflammation and are considered key drivers of HAND pathogenesis (Hellmuth et al., 2015; Zayyad and Spudich, 2015; Saylor et al., 2016).

The infected cells can then release HIV viral proteins into the CNS, including Tat and gp120, which act as extracellular mediators of neurotoxicity, contributing to CNS damage (Wallet et al., 2019). In particular, it was reported that about 70% of the total Tat produced by an HIV-infected cell is extracellularly released during the early phases of cell infection (Ensoli et al., 1990, 1993, 1994, 1997; Chang et al., 1997; Rayne et al., 2010).

HIV gp120 and Tat cause neurodegeneration via mitochondrial dysfunction, synaptodendritic injury, neuroinflammation, and Alzheimer’s disease-like changes, with Tat CSF levels remaining stable at pico- or nanomolar post-cART (Hategan et al., 2019; Darbinian et al., 2020). These mechanisms are the basis of HAND, which can range from mild neurocognitive impairment to severe dementia (Fields et al., 2016). The understanding of the molecular interactions between HIV and neurodegenerative processes is crucial for developing novel therapeutic strategies aimed at preventing or mitigating cognitive decline in this population, given the increasing age of people living with HIV due to successful antiretroviral therapy. Furthermore, these HIV-mediated neurodegenerative processes show convergence with pathological processes typically experienced in classical neurodegenerative diseases, suggesting that Tat and gp120 may be implicated in clinical deterioration and in the aging-related disorders of HIV-infected patients (Canet et al., 2018; Hategan et al., 2019; Mustafa et al., 2024).

The aim of this review is to investigate and elucidate the roles of extracellular Tat and gp120 in neurotoxicity and their direct or indirect contributions to the development of HAND, and to explore potential therapeutic targets to counteract cognitive degeneration in HIV patients.

## 2 Methods

This narrative review was conducted through a comprehensive literature search in major biomedical databases, including PubMed/MEDLINE, Web of Science, Scopus, and Embase.

As a research strategy, Boolean operators were combined with targeted keywords, such as “HIV Tat,” “gp120,” “HAND,” “HIV-associated neurocognitive disorders,” “neurodegeneration,” “Alzheimer’s disease,” “mitochondrial dysfunction,” “neuroinflammation,” “glial activation,” “excitotoxicity,” and their combinations.

To capture recent advances, articles published in the last 21 years (2005–2026) were prioritized. Only studies in English were included, encompassing original research articles, narrative and systematic reviews, preclinical studies (*in vitro* neuronal/glial cultures, animal models), and clinical studies (human cohort studies, postmortem analyses, CSF biomarker data). Non-peer-reviewed studies, case reports, and studies lacking mechanistic insights into Tat/gp120 neurotoxicity were excluded.

The search was conducted independently by the authors, screening titles and abstracts for relevance. To confirm their inclusion, the full texts of potentially eligible articles were assessed. Additional relevant publications were found by manually screening the reference lists of key articles. Given the narrative nature of this review, no formal meta-analysis was performed.

## 3 Structure and roles of Tat

Trans-activator of transcription protein (Tat) is a key factor in the successful transcription of full-length HIV RNA following HIV proviral genome integration into the host cell genome. This protein enhances viral RNA elongation by binding the viral RNA TAR region. Tat gene is encoded by two exons through differential splicing of viral transcripts, resulting in two major distinct isoforms of 86 (Tat_86_) and 101 (Tat_101_) amino acids. The longer isoform is the more common isoform and it is detected in a large array of clinical viral isolates, whereas the shorter form Tat_86_ arises from a single nucleotide change in the second exon, leading to a stop codon that interrupts Tat synthesis at amino acid 86 (Li et al., 2009; Debaisieux et al., 2012).

The first exon encodes for the first 72 amino acids (1–72) and contains the domains involved in the regulation of viral transcription, whereas the second exon encodes for the integrin binding region and is involved in NF-κB activity (amino acids 73–101 or 73–86) (Gotora et al., 2023; Cafaro et al., 2024; Weiling et al., 2025). Exon 1 is highly conserved across different HIV subtypes and contains the major functional domains required for transactivation. Exon 2, on the other hand, exhibits considerable sequence and length variability between different viral clades and encodes the C-terminal region (Guo et al., 2003; Smith et al., 2003; Avraham et al., 2004; Mattson et al., 2005). Tat tolerates substantial sequence variation (up to 40%) without loss of function, thanks to structural constraints in its conserved Zn-finger and core motifs combined with flexibility in flanking regions. Nuclear magnetic resonance studies confirmed that Tat lacks a stable conformation, but it fluctuates between several conformations, giving it the ability to interact with a wide range of host proteins (Shojania and O’Neil, 2006).

**Figure 1:**
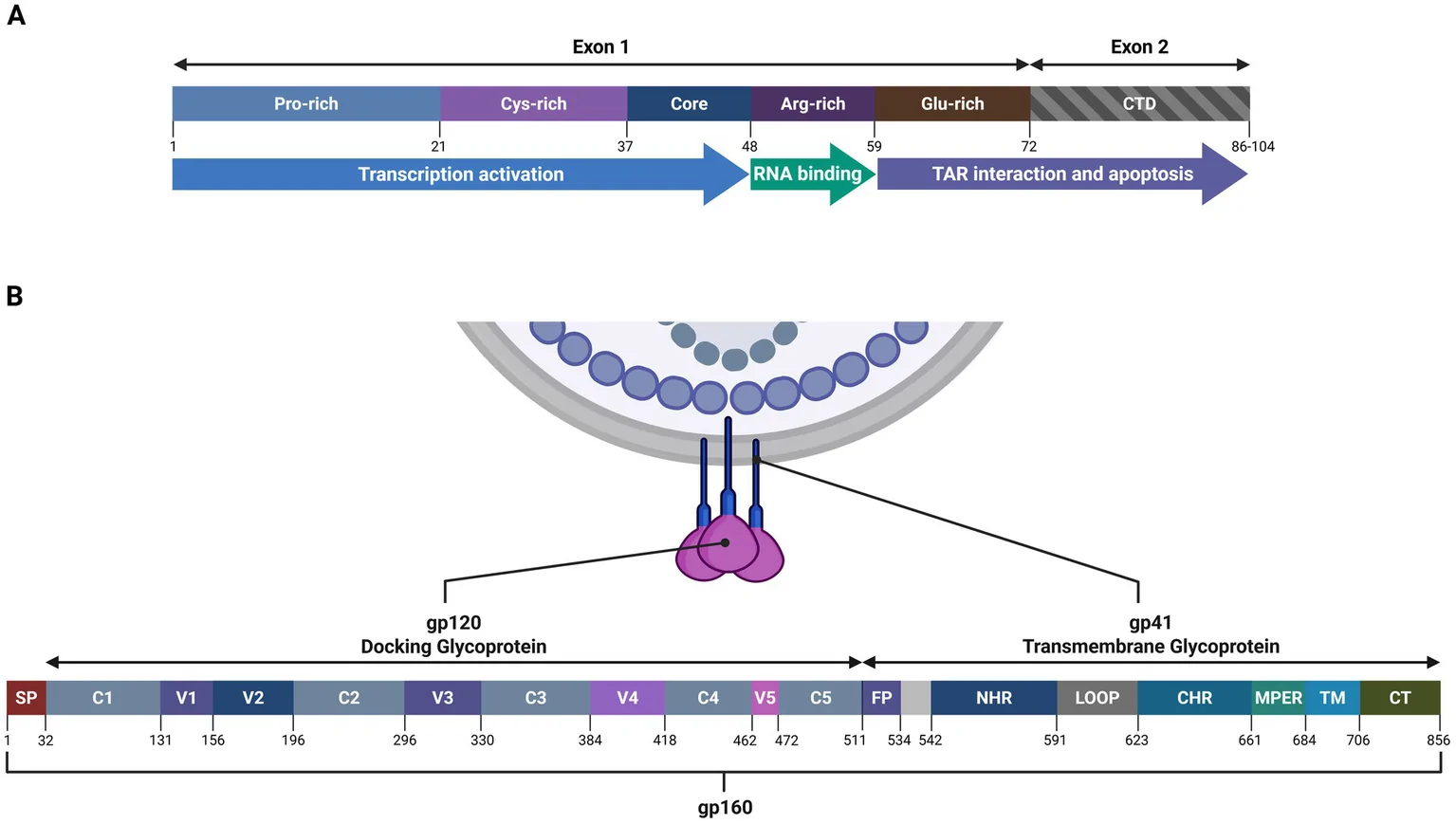
Structure of HIV-1 Tat and gp120 viral proteins. **(A)** Structure of Tat protein and its functional regions. The first five domains, coded by the first exon, are divided into an acidic proline-rich N-terminus (Pro-rich), a cysteine-rich region (Cys-rich), a hydrophobic core (core), an arginine-rich basic domain (Arg-rich), and a glutamine-rich region (Glu-rich); the second exon codifies for a C-terminal domain (CTD). **(B)** Domain structure of the HIV-1 envelope glycoproteins. Precursor gp160 contains the signal peptide (SP), cleaved during translation. gp120 is composed of five relatively conserved domains (C1–C5) interspersed by five variable domains (V1–V5). Instead, the gp41 subunit can be subdivided into the following functional regions: fusion peptide domain (FP), a N-terminal heptad repeat (NHR), a loop region (LOOP), a C-terminal heptad repeat (CHR), a membrane proximal external region (MPER), a transmembrane domain (TM), and a cytoplasmic tail (CT) (created with BioRender.com).

HIV-1 Tat protein is typically organized in several functional domains encoded primarily by exon 1: an N-terminal proline-rich transactivation domain (residues 1–21), a cysteine-rich domain (residues ~22–37) involved in metal coordination, a hydrophobic core (38–48), followed by a basic arginine-rich RNA-binding domain (~49–59) and a glutamine-rich domain (~60–72) while the C-terminal segment derived from exon 2. The arginine-rich domain mediates high-affinity TAR RNA binding, while the cysteine-rich and core regions primarily support the assembly and stability of the Tat-P-TEFb-TAR complex rather than directly contacting TAR on their own (Bayer et al., 1995; Tahirov et al., 2010; Clark et al., 2017). The structural organization of Tat is summarized in Figure 1A.

Tat protein plays prominent roles in the HIV replication cycle and in the deregulation of host cell signaling pathways. After HIV entry into the target cell, Tat plays a key role in viral replication by reactivating RNA polymerase II, promoting a rapid elongation and transcription of HIV genes, and strongly stimulating capping of HIV mRNAs in a co-transcriptional mode (Chiu et al., 2002). It is also known that Tat contributes to the initiation of the reverse transcription (Harrich, 1997), the earliest and most delicate phase after viral entry into the host cell. Tat is also involved in the modulation of the alternative splicing process, for HIV RNAs and host cells RNAs (Bannwarth and Gatignol, 2005; Mueller et al., 2018).

Thanks to the presence of a chemokine-like region, Tat can mimic β-chemokine function to recruit monocytes and macrophages near HIV-producing cells, facilitating simpler activation and infection of these cells (Albini et al., 1998). HIV Tat also alters cytoskeletal structure and protein functions, contributing to host cell deregulation and viral replication (Lopez-Huertas et al., 2010). In addition, it acts by slowing down cell apoptosis by temporarily blocking the Fas pathway (López-Huertas et al., 2013). In antigen-presenting cells (APCs), Tat reduces innate immune activation by decreasing transcription of interferon-stimulated genes and MHC molecules (Howcroft et al., 1993; Kanazawa et al., 2000; López-Huertas et al., 2013; Kukkonen et al., 2014; Cafaro et al., 2024). Tat plays a major role in viral transcription by recruiting the cellular P-TEFb complex (CDK9/cyclin T1) to the TAR stem-loop RNA structure within the HIV long terminal repeat (LTR) promoter. Upon binding, Tat adopts an extended conformation, primarily engaging Cyclin T1 TRM arm through its cysteine-rich and core domains, while its arginine-rich basic domain contacts both TAR RNA and CDK9. These interactions induce key conformational changes in P-TEFb, enhancing its kinase activity and altering substrate specificity. These modifications enable efficient phosphorylation of both Ser2 and Ser5 within the heptad repeats of RNA polymerase II C-terminal domain (CTD). The Tat-P-TEFb interface buries 3,499 Å^2^ of surface area (88% on Cyclin T1, 12% on CDK9), with 37 Tat residues forming a complementary fit (Bayer et al., 1995; Tahirov et al., 2010; Clark et al., 2017). This process potentiates the RNA polymerase II activity, which elongates the nascent viral RNA.

Beyond its canonical role in viral transcription, accumulating evidence indicates that Tat also promotes long-term alterations in host cell homeostasis. In particular, the full-length Tat isoform containing the second exon has been shown to induce a senescence-like phenotype characterized by increased expression of cell-cycle arrest markers (p21CIP1 and p16INK4A), DNA damage marker γH2AX, anti-apoptotic protein BCL-2, and the senescence-associated secretory phenotype (SASP), leading to sustained secretion of pro-inflammatory cytokines (Casanova et al., 2025). Similar observations in microglial cells (Thangaraj et al., 2021) suggest that Tat-induced senescence may contribute to the establishment of a chronically inflamed and prematurely aged CNS microenvironment. In addition, emerging studies indicate that Tat may induce epigenetic changes, including alterations in DNA methylation and gene expression linked to pro-inflammatory and anti-apoptotic pathways (Deinhardt-Emmer et al., 2025), providing a potential mechanism for the long-term persistence of HIV-associated neuroinflammation despite effective antiretroviral therapy.

The biological characteristics of Tat were enriched by its extracellular release from infected cells in large amounts (about 2/3 of the total Tat protein produced by host cells). Tat can stimulate cell growth and viral transactivation by autocrine and paracrine processes. *In vitro,* extracellular Tat binds various cell-surface receptors (Ensoli et al., 1993) and promotes cell adhesion by binding the Tat RGD region to the integrin surface receptors α5β1 and αVβ3 (Barillari et al., 1993; Vogel et al., 1993). In addition, other receptors showed a binding affinity for Tat, including CD26, CCR-2, CCR-3, CD91, and VEGF-R1, expanding the spectrum of biological activities on different cell types (Cafaro et al., 2024).

Tat can affect cell viability, including primary cells (i.e., lymphocytes), and several cell lines, depending on Tat concentration and cell type (Li et al., 1995; King et al., 2006).

In CNS, Tat cationic amphiphilic properties enable efficient, non-receptor-mediated uptake into neurons and glial cells through endocytosis or direct membrane translocation. Once internalized, Tat dysregulates host gene expression, such as by competing with HEXIM1 to unbind P-TEFb from inhibitory 7SK snRNP complexes, while activating pro-apoptotic pathways and modulating immune responses. Tat maintains remarkable stability in CSF, with a half-life of days at concentrations of 10–100 pM, sufficient to activate receptors and undergo BBB transcytosis (Bayer et al., 1995; Tahirov et al., 2010; Clark et al., 2017; Darbinian et al., 2020).

Beyond its essential role in viral replication, Tat remarkable extracellular stability and widespread cellular uptake make it a persistent mediator of CNS injury. These properties are shared, although through distinct molecular interactions, by the second major HIV neurotoxic protein, gp120.

## 4 Structure and roles of gp120

The viral surface glycoprotein of the HIV-1 envelope, gp160, is cleaved into two transmembrane components, gp120 and gp41.

It comprises approximately 856 amino acids and contains a signal peptide (SP) (residues 1–31), which is cleaved during translation. The gp120 subunit is composed by five relatively conserved domains (C1-C5), and five hypervariable loops (V1-V5) that create a crown-like structure masking conserved receptor-binding sites, while glycans like that at Asn262 (99% conserved, GlcNAc₂Man₇ modeled with buried GlcNAc stem in a protein cleft bridging inner/outer domains) stabilize the process of folding by occluding solvent access and interacting with adjacent glycans (e.g., Asn295, Asn332 in the oligomannose patch). The ectodomain of gp120 bears extensive N-linked glycosylation (~50 glycans, molecular mass 20–30 kDa) that forms a protective glycan shield, as visualized in the 4.5 Å crystal structure of a fully glycosylated YU2 gp120 core (PDB: 4RQS) (Kwong et al., 1998; Kong et al., 2015). The protein folds into a heart-shaped core featuring two principal domains: an inner domain (a two-helix/two-strand bundle connected to a five-stranded β-sandwich and V1/V2 stem protrusion) and an outer domain (stacked double β-barrel: proximal barrel formed by a twisted six-stranded mixed β-sheet embracing helix 2 as the seventh stave; distal barrel as a seven-stranded antiparallel β-sheet sharing a common hydrophobic core) linked by a distinct four-stranded bridging sheet minidomain (composed of strands 3, 2, 21, and 20, incorporating V1/V2 stem and 20–21 hairpin elements) (Kwong et al., 1998; Kong et al., 2015). The structural organization of gp120 is illustrated in Figure 1B.

The CD4-binding site, located at residues 365–372 within a prominent Phe43 cavity at the junction of inner/outer domains and bridging sheet, along with coreceptor-binding regions (V3 loop base determining CCR5/CXCR4 tropism), undergoes substantial CD4-induced conformational rearrangements. This rearrangement exposes the bridging sheet for coreceptor docking, triggers gp41-mediated membrane fusion, and creates large interfacial cavities, a 279 Å water-filled buffer lined by variable residues (e.g., Ala281, Ser364, Thr455, Arg469) and a 152 Å hydrophobic Phe43 pocket bordered by conserved hydrophobic residues (Trp112, Val255, Thr257, Glu370, Phe382, Tyr384, Trp427, Met475) (Kwong et al., 1998; Kong et al., 2015).

Glycosylation further shields epitopes (e.g., Asn262 stabilizes the core structure and interacts with oligomannose patch glycans at Asn295, Asn332), modulates receptor interactions and stability, and enables soluble shed gp120 to exert neurotoxic effects through neuronal CXCR4/CCR5/integrin/NMDA receptor engagement, independent of viral entry (Kwong et al., 1998; Kong et al., 2015).

gp41 subunit can be subdivided into several functional regions: the fusion peptide domain (FP), a N-terminal heptad repeat (NHR), a loop region, followed by a C-terminal heptad repeat (CHR), a membrane proximal external region (MPER), a transmembrane domain (TM), and a cytoplasmic tail (CT) (Caillat et al., 2021). Of particular interest, MPER is a highly conserved tryptophan-rich region with a three-chain coiled-coil structure. This region is functionally relevant as alanine replacements of the conserved Trp666-Trp670-Trp672-Trp678-Trp680 cluster block the opening of the fusion pore, probably due to the lipid destabilizing properties of this domain (Bellamy-McIntyre et al., 2007; Cai et al., 2011).

Both Tat and gp120 demonstrated an exceptional CSF stability (gp120 detectable at ng/mL levels post-ART, active at 1–10 nM), facilitated by amphipathic/basic motifs that promote BBB transcytosis and cellular uptake. These properties underpin their persistent extracellular contributions to HIV neuropathogenesis, where Tat Zn-stabilized structural flexibility and gp120 cavity-rich, mobile-layer architecture (featuring an invariant seven-stranded β-sandwich core anchored to gp41 with adaptable interlayer loops and glycan stabilization) enable a balance between conformational plasticity for receptor-triggered viral entry/immune evasion and tight gp41 clamping (UNAIDS, 2026; Bayer et al., 1995; Tahirov et al., 2010; Kong et al., 2015; Clark et al., 2017; Darbinian et al., 2020).

Together, these structural and biochemical properties explain why both Tat and gp120 remain biologically active after their release from infected cells. Their persistence within the CNS provides the mechanistic basis for chronic neuronal exposure and the development of HAND.

## 5 Entry into the CNS and cellular uptake

**Figure 2:**
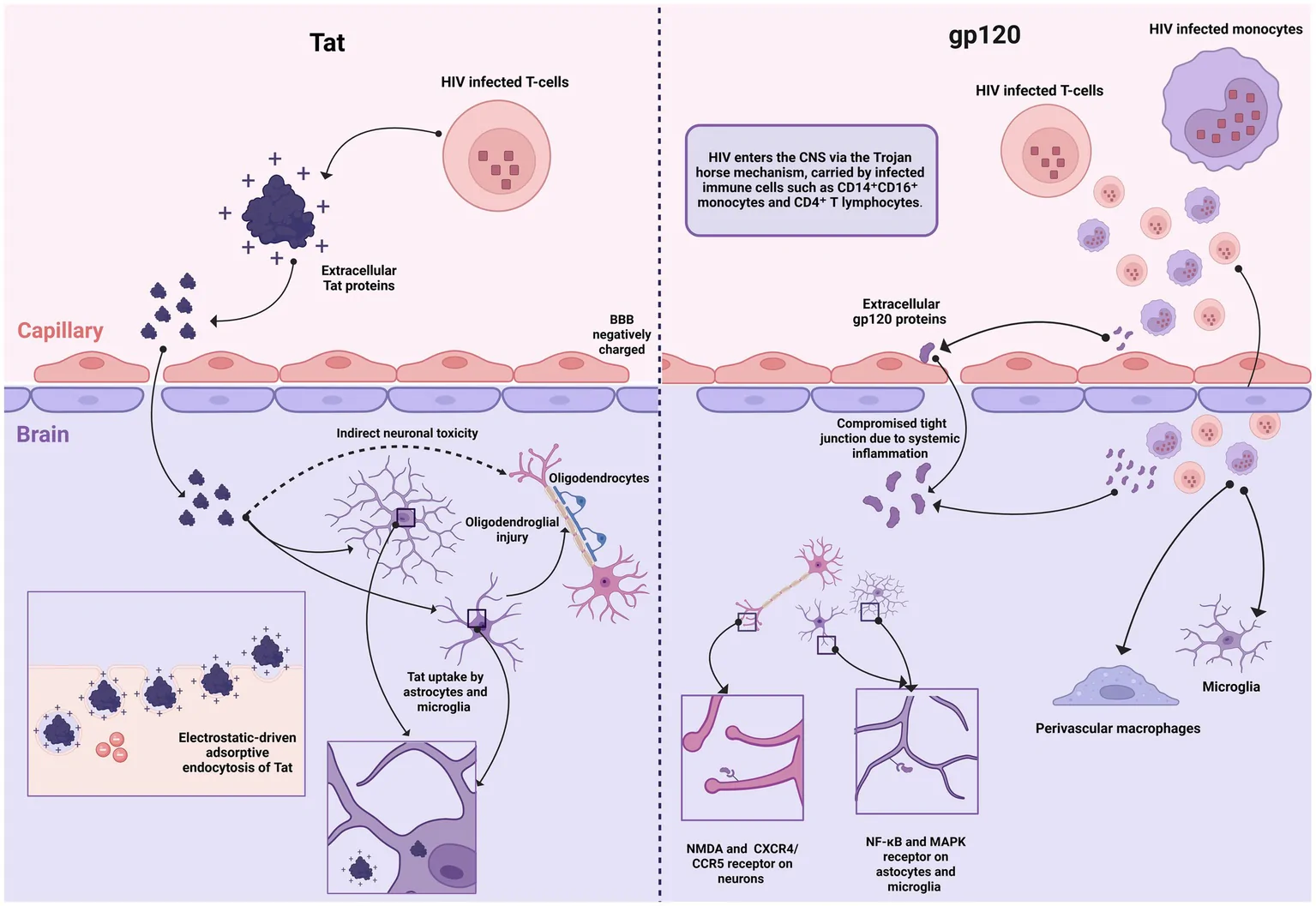
Comparison of the Tat and gp120 proteins penetration into the BBB through the “Trojan horse” mechanism. HIV infection induces an inflammatory state that compromises the integrity of the BBB, facilitating the entry of viral proteins through impacted tight junctions. In this context, Tat penetrates through its high positive charge, due to domains rich in arginine residues, which favor its interaction with the negatively charged endothelial surface, while gp120 crosses the BBB delivered by infected immune cells and through damaged tight junctions (created with BioRender.com).

HIV-1 invasion of the CNS occurs early after systemic infection, typically within days to weeks, although the underlying mechanisms remain poorly understood (Gartner, 2000; Nath, 2002; Bednar et al., 2015). As illustrated in Figure 2, one of the most widely accepted hypotheses is the “Trojan horse” model, whereby infected peripheral CD4 + T-cells and CD14 + CD16 + monocytes transmigrate across the BBB and subsequently infect resident perivascular macrophages and microglial cells (Veenstra et al., 2017; Borrajo et al., 2021; Calado et al., 2024). Thus, durable viral reservoirs are established in perivascular macrophages, microglia, and an astrocyte subset. Therefore, low-level viral transcription persists, leading to continuous production of early viral proteins, including Tat and gp120, as well as lower levels of Nef and Vpr, which remain detectable in CSF and brain tissue despite efficient suppressive antiretroviral therapy (Clark et al., 2017; Wallet et al., 2019). The detection of extracellular Tat in the CSF of patients receiving ART suggests a sustained secretion from latently infected or infected cells. In parallel, gp120 has been detected in CSF, and envelope mRNA has been identified in post-mortem brain tissue, indicating ongoing viral expression within CNS reservoirs, specifically in microglia (Johnson et al., 2013; Henderson et al., 2019; Agbey et al., 2025b). Binding of HIV proteins to neuronal and glial receptors, including CXCR4, CCR5, and TLR2/4/8, together with low-level viral replication in infected macrophages and microglia, activates NF-κB-dependent transcriptional pathways, leading to upregulation of pro-inflammatory cytokines and chemokines and amplification of neuroinflammation (Shah et al., 2016; Kong et al., 2024). HIV-induced endothelial activation and inflammatory mediators, such as TNF-*α*, IL-1β, IL-6, and CCL2, further contribute to BBB dysfunction and, consequently, disruption of tight junction integrity through downregulation of ZO-1 and occludin and increasing paracellular permeability (Osborne et al., 2020; Said and Venketaraman, 2025). This structural impairment facilitates viral entry, promotes infiltration of infected immune cells, particularly CD14 + CD16 + monocytes and CD4 + T cells, and enhances leakage of soluble viral proteins, including Tat and gp120, from the circulation and CSF into the brain parenchyma (Veenstra et al., 2019). Once released into the brain microenvironment, Tat and gp120 reach and enter CNS cells through multiple routes.

Tat readily translocates across cellular membranes through energy-independent transduction and receptor-mediated endocytosis by interactions with low-density lipoprotein receptor-related protein (LRP) and integrins, thereby allowing uptake by neurons, astrocytes, and microglia (Eugenin et al., 2007).

In parallel, gp120 released from virions or infected cells interacts with CD4, CCR5, CXCR4, and other surface molecules expressed on neurons and glial cells (Kovalevich and Langford, 2012a). Through these mechanisms, gp120 accumulates in the CNS parenchyma and CSF, where it interacts with chemokine and glutamatergic receptors to initiate downstream neurotoxic signaling (Jadhav and Nema, 2021; Agbey et al., 2025a). Altogether, these processes ensure the persistent presence of Tat and gp120 within the CNS microenvironment, thereby linking early neuroinvasion and reservoir formation to chronic HIV-associated neurotoxicity despite ART (Saro et al., 2022).

The persistent presence of extracellular Tat and gp120 within the CNS establishes the conditions for sustained neuronal dysfunction. Rather than causing immediate widespread neuronal loss, these viral proteins progressively impair specific neuronal circuits, ultimately contributing to the motor and cognitive manifestations that characterize HAND.

## 6 Tat roles in the dopaminergic system

It is well known that HIV Tat protein plays a crucial role not only in viral replication but also in the neuropathology associated with HIV infection. Among its most significant neurological effects is its impact on the dopaminergic system, a key neural network involved in motivation, reward processing, motor control, and cognition (Gaskill et al., 2017).

Tat is released extracellularly from infected cells, allowing it to affect both infected and uninfected neurons. Within the central nervous system, Tat particularly affects dopaminergic pathways such as the mesolimbic and nigrostriatal systems (Fitting et al., 2015). These pathways, originating in the ventral tegmental area (VTA) and the substantia nigra, are central to reward signaling and motor function. Disruption of these circuits has been implicated in HAND (Gaskill et al., 2017).

One of the primary effects of Tat on the dopaminergic system is the dysregulation of dopamine homeostasis. Tat has been shown to inhibit, through a protein kinase C-dependent mechanism, the dopamine transporter (DAT) (Midde et al., 2012), which is responsible for the reuptake of dopamine from the synaptic cleft. By altering the DAT function, Tat can alter extracellular dopamine levels, leading to abnormal signaling. It was observed that Tat impairs dopamine uptake by altering vesicle dopamine transport. In addition, Tat reduces DAT expression and acts as an allosteric modulator of DAT through a protein–protein interaction. Additionally, Tat promotes oxidative stress and mitochondrial dysfunction in dopaminergic neurons, increasing their vulnerability to damage and apoptosis.

Tat also enhances neuroinflammatory responses by activating microglia and astrocytes (Yang et al., 2025). This inflammatory environment further compromises dopaminergic neurons, which are particularly sensitive to oxidative and inflammatory insults. Over time, such damage may contribute to motor impairments and cognitive deficits observed in people living with HIV.

Moreover, Tat interacts with drugs of abuse, especially psychostimulants like cocaine and methamphetamine (Miao et al., 2025), which also target the dopaminergic system. Studies suggest that Tat can potentiate the neurotoxic effects of these substances, exacerbate dopamine dysregulation, and accelerate neuronal injury. It is shown that Tat and methamphetamine act synergistically by inducing pyroptosis in microglial cells (Miao et al., 2025). In addition to these inflammatory effects, Tat has been shown to synergize with cocaine to induce genome-wide changes in DNA methylation and gene expression, affecting pathways involved in synaptic function, neuroinflammation, and microglial activity. These epigenetic alterations may contribute to persistent neuronal dysfunction and cognitive impairment, providing a potential mechanism through which substance abuse exacerbates HAND (Zhao et al., 2022). These interactions are clinically relevant, as substance use is prevalent in certain HIV-positive populations and may worsen neurological outcomes.

By disrupting dopamine transport, promoting oxidative stress, and amplifying neuroinflammation, Tat contributes to dopaminergic dysfunction and neurodegeneration. Understanding these mechanisms is essential for developing targeted therapies aimed at protecting dopaminergic neurons and mitigating the neurological complications of HIV infection.

Although dopaminergic dysfunction represents one of the most clinically relevant consequences of Tat exposure, it reflects only one component of a broader network of pathogenic events. Both Tat and gp120 activate multiple interconnected molecular pathways that collectively drive neuronal dysfunction and the progressive neurodegeneration underlying HAND.

## 7 Molecular mechanisms of neurotoxicity

In the first weeks after infection, HIV crosses the BBB and stabilizes in the cerebral area, leading to a general inflammatory state (Jia and Brew, 2025). It is well established that the brain-level damage caused by HIV is the main cause of HAND. This is related to a complex interaction between direct damage caused by viral proteins and indirect damage due to a systemic inflammatory state. This process is responsible for oxidative stress, excitotoxicity phenomena, synaptic dysfunction, and progressive neuronal degeneration (Mason et al., 2025).

**Figure 3:**
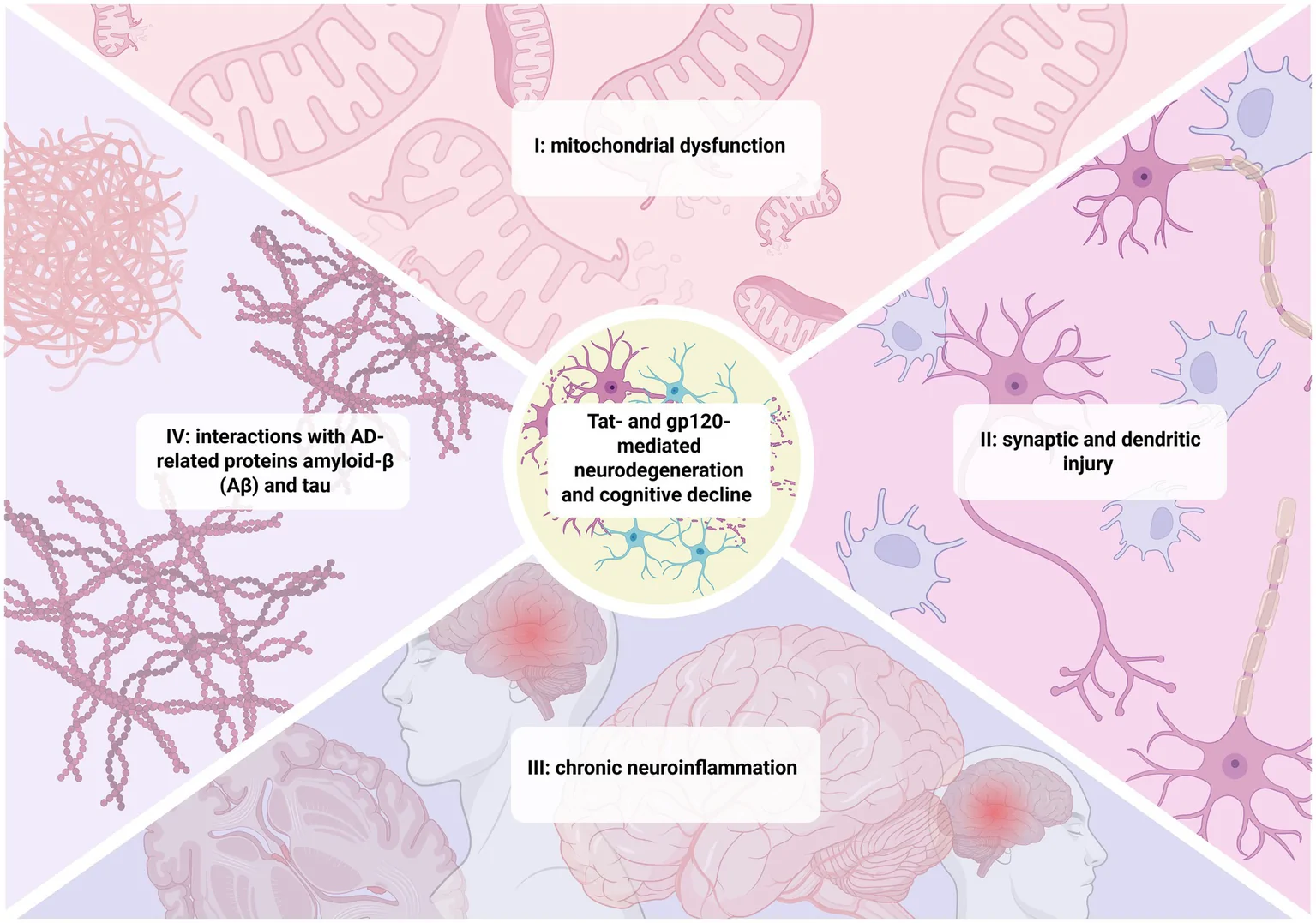
Graphical illustration of the four main mechanisms of neurotoxicity given by Tat and gp120 proteins: mitochondrial dysfunction (I), synaptic/dendritic damage (II), chronic neuroinflammation (III), and interactions with amyloid-β (Aβ), and Tau (IV) (created with BioRender.com).

As illustrated in Figure 3, extracellular Tat and gp120 promote neurodegeneration through four interconnected mechanisms (Rozzi et al., 2017; Wallace, 2022; Kazemeini et al., 2024; Tyagi et al., 2026): (i) mitochondrial dysfunction; (ii) synaptic and dendritic injury; (iii) chronic neuroinflammation; and (iv) interactions with AD-related proteins amyloid-β (Aβ) and Tau. Table 1 outlines the main molecular and cellular events involved in each of the four pathways discussed, together with key supporting references. The interconnection of these mechanisms results in progressive neurodegeneration and cognitive decline in HAND, even with long-term suppressive ART (Canet et al., 2018; Teodorof-Diedrich and Spector, 2018; Hategan et al., 2019; Pansambal and Kurle, 2024; Zhang et al., 2025).

**Table 1:** Principal molecular and cellular events underlying the four pathogenic mechanisms of Tat- and gp120-driven neurodegeneration.

Pathogenic mechanism	Key pathogenic events	References
Mitochondrial dysfunction	Excessive mitochondrial fission, impaired mitochondrial membrane potential, defective mitophagy, ROS production, reduced ATP synthesis, impaired mitochondrial biogenesis	Fields et al. (2016), Teodorof-Diedrich and Spector (2018), Darbinian et al. (2020), Figarola-Centurión et al. (2023), and Shrestha et al. (2023)
Synaptic and dendritic injury	Dendritic simplification, spine loss, excitotoxicity, loss of synaptic proteins (synaptophysin, PSD-95), impaired synaptic plasticity, aberrant synaptic pruning	Cane et al. (2014), Green and Thayer (2016), Green et al. (2019), and Kannan et al. (2022)
Chronic neuroinflammation	Microglial and astrocyte activation, TLR4/NF-κB signaling, NLRP3 inflammasome activation, pro-inflammatory cytokine release (TNF-α, IL-1β, IL-6, IL-18), oxidative stress, persistent ISG expression, complement-mediated synaptic loss, proviral reactivation	Bren et al. (2009), Doyle et al. (2015), Chivero et al. (2017), Roy et al. (2020), Jin et al. (2022), Sreeram et al. (2022), and Kong et al. (2024)
Interaction with Aβ and tau pathology	Aβ overproduction, impaired Aβ clearance, Tau hyperphosphorylation, Tau truncation and aggregation, cytoskeletal destabilization and impaired axonal transport	Ruediger et al. (1997), Alonso et al. (2001), Ehehalt et al. (2003), Amadoro et al. (2006), Kim et al. (2013), Grassi et al. (2020), Rudajev and Novotny (2022), and Pansambal and Kurle (2024)

These pathways do not operate as standalone processes, but rather they are mechanistically linked through bidirectional feedback. Mitochondrial dysfunction represents an early upstream event: impaired mitochondrial dynamics increase reactive oxygen species production and reduce neuronal ATP availability, which in turn compromises synaptic integrity and activates microglial stress responses, initiating neuroinflammation (Fields et al., 2016; Teodorof-Diedrich and Spector, 2018). Conversely, chronic neuroinflammation amplifies mitochondrial injury through sustained cytokine-driven oxidative stress, creating a self-reinforcing cycle (Yang et al., 2025). Synaptodendritic injury and AD-related protein dysregulation emerge downstream of both processes: Aβ accumulation and Tau hyperphosphorylation are promoted by oxidative stress, neuroinflammatory mediators, and impaired proteostasis secondary to mitochondrial failure (Hategan et al., 2019).

### 7.1 Mitochondrial dysfunction

Tat and gp120 directly perturb mitochondrial structure and function in CNS neurons, leading to bioenergetic failure, oxidative stress, and defective mitophagy (Fields et al., 2016; Hu et al., 2017).

Experimental models indicate that, once internalized into neurons, Tat and gp120 significantly impact mitochondrial turnover. The recruitment of dynamin-related protein 1 (DRP1) from the cytosol to the outer mitochondrial membrane is facilitated by these viral proteins, which results in DRP1 oligomerization and mitochondrial constriction, leading to excessive mitochondrial fission (Fields et al., 2016; Hu et al., 2017). DRP1 is a cytosolic GTPase that controls mitochondrial fission and contributes to the dynamic balance between fission and fusion required for mitochondrial quality control and metabolic adaptation. However, if DRP1 is excessively activated or incorrectly located, it can cause uncontrolled fission. This results in a fragmented mitochondrial structure, which features small, dot-like organelles. These changes lead to lower energy efficiency and a higher risk of functional decline (Hu et al., 2017).

In this context, the fragmented mitochondria with depolarized membranes are marked for removal via mitophagy. However, exposure to Tat and gp120 viral proteins leads to the accumulation of partially processed mitophagosomes, suggesting that autophagic degradation stops before completion (Teodorof-Diedrich and Spector, 2018). The simultaneous presence of fragmented, depolarized mitochondria and incompletely cleared mitophagy vesicles disrupts autophagic flux, resulting in damaged mitochondria persisting in neurons, generating reactive oxygen species, and amplifying vulnerability to further damage (Darbinian et al., 2020). These changes have been linked to decreased synaptic vesicle numbers and memory impairments (Fields et al., 2016; Teodorof-Diedrich and Spector, 2018).

This phenotype is induced by upstream receptor-mediated and transcriptional changes. The binding of gp120 to CXCR4/CCR5 and glutamatergic receptors, especially N-methyl-D-aspartate receptors (NMDARs), results in excess calcium influx into the neuronal cytosol (Valeria Catani et al., 2000; Gorska and Eugenin, 2020). The calcium load is initially buffered by mitochondria until mitochondrial overload triggers opening of the mitochondrial permeability transition pore (mPTP) (Rozzi et al., 2017). The opening of mPTP leads to the dissipation of membrane potential, inhibition of ATP synthesis, and the release of pro-apoptotic factors such as cytochrome c (Roggero et al., 2001; Lecoeur et al., 2012).

In parallel, Tat and gp120 alter nuclear transcriptional programs by reducing cAMP Response Element-Binding protein (CREB) activation and downstream expression of the master regulator of mitochondrial biogenesis, PGC-1α (Teodorof-Diedrich and Spector, 2018; Figarola-Centurión et al., 2023), thereby impairing the generation, maintenance, and appropriate axonal targeting of healthy mitochondria. In neurons with particularly high energetic and synaptic demands, this combination of calcium-driven acute mitochondrial injury and chronic suppression of biogenesis and trafficking results in a progressive depletion of functional mitochondria at synapses, increased oxidative stress, and heightened vulnerability to synaptic failure and cognitive impairment (Shrestha et al., 2022, 2023).

In addition, intracellular Tat has been reported to impair mitochondrial function by reducing mtDNA transcription, altering mitochondrial dynamics, and inducing compensatory mitochondrial biogenesis, with stronger effects observed for the full-length Tat isoform containing the second exon, highlighting an additional contribution of this region to mitochondrial dysfunction (Rodríguez-Mora et al., 2015).

### 7.2 Synaptic and dendritic injury

Synaptodendritic damage is a key element of cognitive impairment in HAND (Ru and Tang, 2017; Irollo et al., 2021). The disruption of dendritic spines and synaptic contacts between neurons leads to measurable deficits in cognitive performance, as these structures are essential for signal integration and memory formation. Tat/gp120 exposure causes sustained excitotoxic stress by increasing extracellular glutamate levels and dysregulating its clearance. gp120 enhances glutamate release and upregulates NMDARs expression, while Tat impairs astrocytic glutamate uptake, leading to chronic excitotoxic stimulation at the synapse (Green and Thayer, 2016). This sustained NMDAR activation perturbs calcium homeostasis, triggers downstream signaling cascades that destabilize synaptic architecture and promote dendritic spine loss, and contributes to the mitochondrial calcium overload, as previously described.

To counteract the imbalance between excitatory and inhibitory synaptic transmission induced by calcium channel dysregulation, neurons engage a homeostatic response known as synaptic scaling. In recent HAND models, the HIV Tat has been shown to activate NMDA receptors, triggering downstream Akt signaling and subsequent activation of the E3 ubiquitin ligase Mdm2 (Green and Thayer, 2016). This pathway results in a paradoxical homeostatic mechanism in which Tat-induced NMDA receptor overactivation ultimately leads to a compensatory reduction in synaptic NMDA receptor levels. Similarly, gp120 increases Mdm2 expression in cortical neurons, indicating that both viral proteins converge on this regulatory axis (Green et al., 2019). Through Mdm2, Tat and gp120 decrease the levels of key pre- and postsynaptic proteins, including synaptophysin and the scaffolding molecule postsynaptic density protein-95 (PSD-95) (Cane et al., 2014; Green et al., 2019). The loss of these structural components weakens dendritic spines in cortical and hippocampal neurons. This leads to decreased excitatory synapse density and disrupts glutamatergic transmission. Although this decrease in excitatory transmission aligns with a protective synaptic scaling response, its persistence likely contributes to long-term synaptic vulnerability and cognitive impairment, as observed in experimental models of HAND (Irollo et al., 2021). Beyond their effects on excitatory synapse stability, Tat and gp120 also impair synaptic plasticity by disrupting neurotrophin signaling and cytoskeletal organization (Avdoshina and Mocchetti, 2022). Recent evidence indicates that gp120 can shift the balance of neurotrophin signaling toward pro-brain-derived neurotrophic factor (pro-BDNF) and the p75 neurotrophin receptor (p75^NTR^), which preferentially engages NR2B-containing NMDA receptors and promotes long-term depression (LTD) (Woo et al., 2005). gp120 reduces the furin levels, the protease that converts pro-BDNF into its mature form, and increases p75^NTR^ expression in vulnerable neuronal populations (Yang et al., 2014; Speidell et al., 2020). The build-up of pro-BDNF enhances LTD-related NR2B modulation and decreases dendritic complexity and spine density via p75^NTR^ activation. These effects are attenuated in gp120-transgenic mice with reduced p75^NTR^ expression (Speidell et al., 2019).

Additionally, Tat contributes to synaptic instability through complementary mechanisms. It interferes with actin remodeling and postsynaptic scaffolding, and weakens the structural integrity of dendritic spines (Hu et al., 2020). Moreover, microglia chronic exposure to Tat promotes the release of extracellular vesicles enriched in inflammatory mediators and complement components, thereby enhancing aberrant synaptic pruning (Kannan et al., 2022). Together, these pathways lead to synaptodendritic damage through a mix of excitotoxic stress, lack of neurotrophic support, and microglia-mediated synapse removal. Such multifaceted disruption of synaptic homeostasis likely underlies the persistence of cognitive dysfunction in people with HIV, even in the context of effective viral suppression (Avdoshina and Mocchetti, 2022).

### 7.3 Chronic neuroinflammation

Chronic neuroinflammation represents a persistent, low-grade activation of resident brain immune cells that sustains neuronal damage in HAND, even under effective antiretroviral treatment (Kong et al., 2024). In contrast to acute inflammation, this state involves prolonged signaling from Tat and gp120, which maintain microglia, astrocytes, and brain endothelial cells in a continuously activated state (Chivero et al., 2017). The initiation of intracellular cascades that lead to inflammation occurs when Tat and gp120 bind to specific surface receptors on these cells, such as chemokine receptors CCR5 and CXCR4, which are typically associated with immune cell migration. Additionally, Tat and gp120 are recognized by pattern recognition receptors such as Toll-like receptors (TLRs) on microglia and astrocytes. The activation of TLR4 on microglia, via a cascade mechanism, leads to NF-κB activation, which upregulates genes encoding pro-inflammatory cytokines, including tumor necrosis factor-alpha (TNF-*α*), interleukin-1 beta (IL-1β), and interleukin-6 (IL-6). Recent evidence suggests that this mechanism is microglia-specific, since astrocytes, which poorly express CCR5, are slightly susceptible to HIV infection (Kong et al., 2024).

A critical amplifier is the NLRP3 inflammasome, a multiprotein complex in microglia that plays a key role in pathogen detection, and its activation leads to production and secretion of IL-1β and IL-18. Recent findings show that Tat activates NLRP3 inflammasome in microglia in both dose- and time-dependent manner (Chivero et al., 2017), and it is able to promote NLRP3 inflammasome priming in microglia by suppressing the expression of miR-223, a post-transcriptional repressor that normally limits NLRP3 translation (Chivero et al., 2017). By removing this inhibitory control, Tat increases the availability of NLRP3 and sensitizes microglia to later activation signals, amplifying their pro-inflammatory potential (Li et al., 2020). In addition to Tat-mediated priming of the NLRP3 inflammasome, gp120 also contributes to inflammasome activation in microglia (He et al., 2020). gp120 stimulates the Kv1.3 potassium channel through CXCR4, promoting K^+^ efflux. This process is a known trigger for NLRP3 assembly and caspase-1 activation. Therefore, Tat and gp120 influence distinct yet connected steps of NLRP3 activation, enhancing microglial inflammatory responses in HAND (He et al., 2020).

Moreover, Tat induces activation signals via mitochondrial stress, which releases damage-associated molecular patterns, such as reactive oxygen species and mitochondrial DNA (Yang et al., 2025). Microglial mitochondrial dysfunction thus creates a feed-forward loop in which viral proteins amplify inflammasome activation, cytokine release, and further mitochondrial injury, establishing a self-reinforcing inflammatory state that can persist even under systemic viral suppression. This persistence is further sustained by the bidirectional relationship between chronic neuroinflammation and HIV latency in CNS reservoirs. Long-lived latently infected microglia can sustain low levels of Tat and gp120, maintaining microglial reactivity. Conversely, pro-inflammatory cytokines generated during neuroinflammation (particularly TNF-α and IL-1β) activate NF-κB in these same cells and can trigger proviral reactivation, renew Tat and gp120 production, and sustain microglial reactivity (Jin et al., 2022; Sreeram et al., 2022). This reactivation is also governed by neuronal integrity: healthy neurons actively suppress HIV transcription in latently infected microglia through homeostatic signals, including CX3CL1 and CD200, whereas damaged neurons lose this suppressive capacity and can directly induce proviral reactivation (Alvarez-Carbonell et al., 2019). This intermittent reservoir reactivation is refractory to ART, since antiretroviral drugs do not inhibit HIV transcription or latency reversal (Sreeram et al., 2022). Beyond acting as neurotoxic mediators, Tat and gp120 may also influence reservoir dynamics within the CNS. Tat is the principal viral transactivator required for efficient HIV transcription and promotes latency reversal by recruiting of the P-TEFb complex to the viral LTR (Mbonye and Karn, 2017). In parallel, gp120-induced inflammatory signaling, including NF-κB activation in glial cells, may create a permissive environment for episodic proviral reactivation (Bren et al., 2009). Consequently, Tat and gp120 act as both products of reservoir reactivation and as amplifiers of the inflammatory environment that favors the continued HIV persistence within the CNS.

In addition to those reservoir-driven inflammatory mechanisms, chronic IFN-I signaling has emerged as an important mechanism that may contribute to HAND-associated neuroinflammation and neurodegeneration. While acute IFN-I responses are essential for antiviral defense, persistent activation of IFN-I pathways during chronic HIV infection promotes immune hyperactivation, T-cell exhaustion, and sustained expression of interferon-stimulated genes (ISGs), even in the presence of effective antiretroviral therapy (Doyle et al., 2015; Zhen et al., 2016). Within the CNS, IFN-I signaling activates microglia and drives neuroinflammation through a complement-dependent mechanism: IFN-β directly induces C3 expression in astrocytes and promotes microglial engulfment of synaptic terminals, leading to selective postsynaptic density loss and synaptic dysfunction. Importantly, IFN receptor blockade is sufficient to rescue synapse loss in models of amyloid pathology, demonstrating the causal role of IFN-I in this neurodegenerative process (Roy et al., 2020). Furthermore, chronic IFN-I signaling has been associated with cellular senescence and age-related cognitive decline (Baruch et al., 2014; De Cecco et al., 2019), and ISG expression in the human brain has been positively correlated with both clinical dementia severity and Alzheimer’s disease-related neuropathology (Roy et al., 2020). This evidence suggests that persistent IFN-I activation may represent an additional mechanism linking HIV-associated chronic neuroinflammation and HAND pathogenesis to broader neurodegenerative processes.

### 7.4 Interaction of tat and gp120 with Alzheimer-related proteins, amyloid-β and tau

Tat and gp120 interact with Alzheimer’s disease (AD)-related pathways by accelerating Aβ accumulation, Tau hyperphosphorylation, and formation of hybrid HAND-AD phenotypes in aging people living with HIV. In this context, the hybrid HAND-AD phenotype denotes a virus-driven neurodegenerative condition in which Tat and gp120 directly engage AD-related molecular cascades, generating AD-like neuropathology through mechanisms. Such mechanisms are independent of the aging- and genetics-driven processes underlying sporadic AD, and the hybrid phenotype should therefore not be interpreted as mere co-occurrence of two independent diseases in the same individual (Mustafa et al., 2024).

Aβ is an intrinsically disordered peptide of 36–43 amino acids generated through the amyloidogenic processing of the amyloid precursor protein (APP) by β- and γ-secretases. The predominant isoforms, Aβ1-40 and Aβ1-42, share a tripartite structural organization comprising a flexible, hydrophilic N-terminal region that lacks stable secondary structure in solution; a central hydrophobic core (residues 17–21) that drives self-assembly through its strong propensity to form β-sheet interactions; and a highly hydrophobic C-terminal segment, particularly extended in Aβ1-42, which stabilizes intermolecular contacts and markedly enhances aggregation and neurotoxicity (Coles et al., 1998; Alonso et al., 2001; Chen et al., 2017).

Structural studies indicate that monomeric Aβ remains largely unstructured (as reviewed in Hubin et al., 2014). However, during oligomerization, it adopts β-strand conformations. This enables the formation of protofibrils and mature fibrils characterized by the canonical cross-β architecture, with β-strands oriented perpendicular to the fibril axis. Collectively, the intrinsically unstructured nature of the monomer, its hydrophobic aggregation-prone core, and its highly amyloidogenic C-terminal region cooperate to drive the formation of neurotoxic oligomers and the fibrillar deposits characteristic of Alzheimer’s disease (Chen et al., 2017; Bhopatkar and Kayed, 2023).

Tau is a highly soluble protein and is associated with microtubules. It is encoded by the MAPT gene and exists in six major forms in the central nervous system, generated through alternative splicing. Structural analyses reveal that monomeric Tau is flexible and mainly unstructured. It lacks a stable tertiary structure and adopts dynamic conformations in solution (Avila et al., 2016). Tau is the main microtubule-associated protein in mature neurons; it binds tubulin, promotes microtubule polymerization, and stabilizes the cytoskeletal network. This activity is carefully controlled by the state of Tau phosphorylation. When phosphorylation increases, Tau ability to support microtubule assembly decreases. In AD, Tau becomes three- to four-fold more hyperphosphorylated than in the healthy adult brain, a modification that markedly diminishes its microtubule-stabilizing function and promotes its self-assembly into paired helical filaments and straight filaments (Hategan et al., 2019). Hyperphosphorylated Tau not only loses its normal function but also traps regular Tau and other microtubule-associated proteins, worsening cytoskeletal instability. Additionally, some Tau molecules in the Alzheimer’s brain undergo proteolytic truncation, increasing their tendency to aggregate and speeding up filament formation. During oligomerization, Tau monomers shift from diverse shapes to conformations rich in β-strands (Naseri et al., 2019). This results in amyloid filaments with a cross-β structure, typical of tauopathies, including Alzheimer.

At the ultrastructural level, Tau filaments exhibit a characteristic organization in which the ordered β-sheet backbone of the fibril is surrounded by the remaining unstructured segments of the polypeptide chain. These disordered regions create a dynamic, solvent-exposed “fuzzy coat” that surrounds the fibril core. This coat helps stabilize the filaments, affects intermolecular interactions, and influences the distinct behavior of Tau aggregates in various tauopathies (Zeng et al., 2021). Tat interacts directly with APP at the surface of neuronal cells. It binds to APP’s extracellular domain and changes its membrane movement. This process helps relocate APP into lipid rafts rich in cholesterol and sphingolipids, where β- and γ-secretases are concentrated (Ehehalt et al., 2003; Grassi et al., 2020). As observed in primary neurons and human neuroblastoma cells (Rudajev and Novotny, 2022), Tat enhances BACE1 catalytic activity and γ-secretase membrane recruitment through raft-dependent signaling and oxidative stress, markedly increasing Aβ1-42 generation, which is the most aggregation-prone amyloid species (Kim et al., 2013).

In parallel with Tat, gp120 further amplifies amyloidogenic APP processing through complementary mechanisms. gp120 engages neuronal chemokine receptors, CXCR4 and CCR5, triggering intracellular signaling cascades that increase intracellular calcium, activate MAPK pathways, and promote oxidative stress, all of which are known to modulate APP cleavage. These signaling events increase the expression and activity of BACE1 and enhance γ-secretase–dependent processing, shifting APP metabolism toward the amyloidogenic pathway (Rudajev and Novotny, 2022). In neuronal cell cultures, gp120 exposure substantially increases Aβ production, particularly Aβ1-42, and this effect is potentiated by Tat, indicating a synergistic disruption of APP homeostasis. Beyond its impact on APP processing, gp120 perturbs lipid raft composition and membrane cholesterol distribution, thereby indirectly facilitating the recruitment of APP and secretases into raft microdomains (Rudajev and Novotny, 2022).

Beyond promoting Aβ overproduction, Tat and gp120 impair Aβ clearance, further exacerbating amyloid accumulation. Tat disrupts low-density lipoprotein receptor-related protein 1 (LRP1), mediated Aβ efflux across the BBB, thereby reducing Aβ transport from the brain to the periphery (Liu et al., 2000). Additionally, gp120 downregulates key Aβ-degrading enzymes, including neprilysin and insulin-degrading enzyme (IDE), thereby diminishing proteolytic clearance (Rempel and Pulliam, 2005). Chronic neuroinflammation induced by Tat- and gp120-dependent signaling promotes a dysfunctional and pro-inflammatory state in microglia, leading to reduced phagocytic capacity, which further contributes to impaired Aβ removal (Pansambal and Kurle, 2024).

Through Tat and gp120 interactions with signaling pathways that regulate Tau phosphorylation, microtubule binding, and proteostatic turnover, they can also significantly influence Tau homeostasis. Tat activates multiple kinase cascades, among them cyclin-dependent kinase 5 (CDK5), glycogen synthase kinase-3β (GSK3β), Extracellular signal-regulated Kinase 1/2 (ERK1/2), and p38 MAPK, through calcium dysregulation, oxidative stress, and NMDA receptor potentiation, leading to hyperphosphorylation of Tau at AD-relevant epitopes (Kimura et al., 2014; Dunning et al., 2016; Fields et al., 2016).

This hyperphosphorylated Tau has a lower affinity for microtubules. This change leads to cytoskeletal destabilization, impaired axonal transport, and increased levels of soluble, aggregation-prone Tau species (Alonso et al., 2001). Tat interferes with phosphatase activity, particularly PP2A, further shifting the phosphorylation equilibrium toward pathogenic Tau states (Ruediger et al., 1997). gp120 amplifies these effects by engaging CXCR4 and CCR5, triggering downstream activation of GSK3β and CDK5, and inducing mitochondrial dysfunction and ROS production. These changes improve Tau phosphorylation and cleavage (Melov et al., 2007; Teodorof-Diedrich and Spector, 2018; Avdoshina and Mocchetti, 2022). Both Tat and gp120 promote calpain activation, generating N-terminally truncated Tau fragments with heightened aggregation propensity and neurotoxicity. This calpain-dependent pathway mirrors the excitotoxic mechanism described in tauopathy models, where calcium influx through overactivated NMDA receptors drives sustained ERK1/2 activation, CREB shut-off, and proteolytic cleavage of Tau. Activation of calpain leads to the production of a 17 kDa N-terminal Tau fragment and additional truncated species that exhibit enhanced neurotoxicity and aggregation propensity (Amadoro et al., 2006). These fragments exacerbate microtubule destabilization, amplify NMDA receptor overactivation, and sustain a feed-forward loop of calcium dysregulation and proteolysis. Through this mechanism, Tat and gp120 not only promote Tau hyperphosphorylation but also accelerate the formation of toxic Tau fragments, thereby compromising axonal integrity, resembling key features of excitotoxic tauopathy (Amadoro et al., 2006).

Collectively, these alterations destabilize axonal stability and drive the formation of hyperphosphorylated, truncated Tau species, characteristic of neurodegenerative tauopathies, thereby providing a mechanistic link between chronic HIV protein exposure and AD-like neurodegeneration in HAND.

## 8 Clinical and preclinical evidence of HAND and convergence with AD

Despite advances in antiretroviral therapy, HAND remains prevalent, while age was not significantly associated in early meta-analyses (Wei et al., 2020). Recent studies report higher rates of symptomatic HAND in aging people living with HIV (PLWH) (>50 years), potentially driven by accumulating comorbidities and persistent neuroinflammation (Kinai et al., 2017; Flatt et al., 2023). Age-related impairment of brain waste-clearance mechanisms, including the glymphatic and meningeal lymphatic systems (Benveniste et al., 2019; Nedergaard and Goldman, 2020), may further contribute to this increased vulnerability by reducing the clearance of extracellular viral proteins such as Tat and gp120, thereby prolonging their persistence and neurotoxic effects within the CNS (Tice et al., 2020). This mechanism may be particularly relevant given the long-term survival of PLWH receiving effective cART, in whom viral proteins may continue to exert pathogenic effects despite suppressed viral replication.

According to Frascati criteria, HAND can be categorized into three types based on disease severity: asymptomatic neurocognitive impairment (ANI), mild neurocognitive disorder (MND), and HIV-associated dementia (HAD), characterized by deficits in executive function, processing speed, and episodic memory, distinct from the amnesia-dominant profile of AD. Longitudinal cohort studies demonstrate that while severe HAD has declined post-cART, milder forms persist, driven by chronic neuroinflammation and persistent viral reservoirs rather than active replication (Heaton et al., 2011).

CSF biomarkers offer important insights into the nature of neuronal injury in HAND and its potential overlap with AD. Neurofilament light chain, a sensitive marker of axonal degeneration, is consistently elevated in PLWH with cognitive impairment and correlates with white-matter damage and global cognitive decline (de Almeida et al., 2018; Mackiewicz et al., 2019).

Tau biomarkers show a more nuanced pattern: total Tau (t-Tau) and phosphorylated Tau (p-Tau181) can be increased in HAND, particularly in older individuals; however, the p-Tau/t-Tau ratio is typically lower than in AD, suggesting a distinct phosphorylation pattern rather than a canonical AD-like tauopathy. Amyloid markers also reveal partial convergence: CSF Aβ42 is frequently reduced in aging HAND cohorts, mirroring AD, although Aβ42/Aβ40 ratios provide weaker diagnostic discrimination, indicating that amyloid dysregulation in HAND may be mechanistically different from classical amyloidogenic processes in AD (de Almeida et al., 2018; Mackiewicz et al., 2019). Together, these biomarker profiles indicate that HAND and AD exhibit partial but biologically relevant overlap while retaining distinct signatures, highlighting the need for multimodal approaches to differentiate HAND from coexisting AD in aging PLWH.

Neuroimaging findings further support the view that HAND and AD share partially overlapping patterns of neurodegeneration while remaining distinguishable entities. Structural and functional MRI studies commonly report subcortical and frontostriatal involvement in HIV-infected patients, together with alterations in large-scale cognitive networks (Milanini and Valcour, 2017; Mackiewicz et al., 2019).

Molecular PET imaging with β-amyloid ligands has revealed increased or atypical binding in subsets of older HIV patients with cognitive impairment (Mohamed et al., 2020; Vera et al., 2021). For instance, [18F]florbetaben PET showed positivity in 20% of HIV patients with cognitive symptoms, with elevated accumulation of Tau and Aβ in AD-typical regions, including the posterior cingulate, superior temporal, and frontal cortices (Vera et al., 2021), while [18F]florbetaben PET confirmed central amyloid deposition in a HIV-infected 71-year-old with mixed dementia features (Turner et al., 2016). These findings suggest that HIV-related neurodegeneration may intersect with AD-associated proteinopathies in vulnerable individuals. However, these Aβ PET signals often display non-canonical spatial patterns, reinforcing evidence that HAND and AD share biological intersections, including upregulated BACE1-mediated APP cleavage, without being mechanistically identical (Rubin et al., 2019).

The persistent detection of HIV proteins such as Tat and gp120 in the CSF of ART-suppressed PLWH provides a mechanistic link to ongoing neuronal injury and associated neuroimaging abnormalities (e.g., subcortical atrophy, white matter disruption) in aging individuals, underscoring the need for HAND-specific biomarkers to differentiate HIV-driven neurodegeneration from comorbid AD (Marino et al., 2020).

Preclinical models elucidate mechanisms of neurotoxicity mediated by Tat and gp120 that resemble HAND pathology and AD convergence (Giunta et al., 2009; Kim et al., 2013; Hategan et al., 2019). Tat transgenic mice with inducible GFAP-driven expression exhibit synaptic loss, dendritic simplification, and cognitive deficits in Morris water maze tasks that mirror mild HAND phenotypes (Bruce-Keller et al., 2008; Fitting et al., 2013; Leibrand et al., 2017; Hategan et al., 2019). These models demonstrate the disruption of BBB via a permeability test, like Na-F/HRP leakage, and microglial activation in Tat-transgenic mice; HIV-1 promotes Aβ accumulation via BBB-derived extracellular vesicles (András et al., 2017; Leibrand et al., 2017). HIV-1 Tat reduces apical dendritic spine density in CA1 pyramidal neurons, suppresses long-term potentiation, and alters synaptic proteins with decreased synaptotagmin-2 and increased gephyrin, without inducing CA1 pyramidal neuron death (Fitting et al., 2013). Intra-CP injection of gp120 induces dose- and time-dependent NMDAR hypersensitivity and excitotoxicity through extrasynaptic NR2B receptors, increasing excitatory post-synaptic current (EPSC), and mini EPSC (mEPSC) frequency, and intracellular calcium via CXCR4 signaling without affecting AMPA receptors, alongside neuroinflammation characterized by microglial and astrocytic activation, MIP-1α production, and neuron loss (Louboutin et al., 2010; Zhou et al., 2017). Downstream mechanisms include the release of extracellular vesicles (ECVs) from the BBB caused by HIV-1. These ECVs are rich in amyloid-β. Astrocytes and pericytes then take up these ECVs. This process promotes the perivascular amyloid deposition seen in the brains of HIV-positive individuals (András et al., 2017). In parallel, HIV-associated activation of the microglial NLRP3 inflammasome-potentially triggered by gp120 or Aβ-drives Tau hyperphosphorylation through IL-1β-dependent signaling and kinases such as CaMKIIα (Thr286) and GSK-3β (Tyr216). These effects are attenuated in NLRP3-deficient models, supporting the existence of an inflammatory–Tau axis (Ising et al., 2019). Complementarily, Tat directly interacts with amyloid precursor protein through its cysteine-rich domain, recruiting APP to lipid rafts and enhancing β-secretase cleavage, elevating β-CTF five-fold and Aβ42 levels (Kim et al., 2013). PSAPP mice, transgenic mice that develop amyloid plaques and cognitive deficits early, confirm that Tat accelerates Aβ burden and Tau tangle formation through Aβ-Tat complexes and microglial activation, with Tat/gp120 activating NMDAR/calpain pathway to produce toxic Tau fragments, increasing ERK1/2, and decreasing CREB activity (Giunta et al., 2009; Kim et al., 2013; Hategan et al., 2019). Converging evidence underscores the contribution of Tat and gp120 to HAND persistence. Tat induces synaptic loss, Aβ accumulation, Tau phosphorylation, and correlates with memory deficits. Intrahippocampal administration of gp120 causes NMDAR excitotoxicity and BBB disruption, leading to learning deficits. Consistently, PSAPP/Tat double-transgenic mice exhibit accelerated plaque and tangle formation along with an exacerbated decline in cognitive performance (Giunta et al., 2009; Louboutin et al., 2010; Zhou et al., 2017; Hategan et al., 2019). These models validate Tat/gp120 contributions to HAND persistence and AD-like pathology.

Building on the clinical and preclinical evidence reviewed earlier, this final section synthesizes neuropathological and biomarker data underscoring the convergence of HAND with AD in aging HIV-infected.

These findings redefine HAND not merely as an HIV-specific entity but as a strong accelerator of AD neuropathologic change (ADNC), needing integrated diagnostic frameworks (Mackiewicz et al., 2019; Zaikos et al., 2025).

Autopsies from community-based cohorts of virally suppressed HIV-patients aged >60 years reveal comorbid ADNC, including Aβ plaques, Tau tangles, and TDP-43 aggregates, although with a predominantly subcortical distribution distinct from canonical cortico-limbic AD patterns (Zaikos et al., 2025). Tau PET imaging reveals positive signals in older individuals with HAND, particularly within mesiotemporal regions that overlap with primary age-related tauopathy and early AD*. In vitro* studies further demonstrate the high affinity of MK-6240 (Kd 0.15–0.32 nM), a highly specific PET tracer for visualize and quantify neurofibrillary tangles of Tau protein in the brain, for AD-like paired helical filament Tau compared with non-AD tauopathies (Malarte et al., 2021; Gérard et al., 2025). Together, these patterns indicate accelerated proteopathy in HIV-driven neurodegeneration (Mackiewicz et al., 2019).

Cerebrospinal fluid analyses reveal elevated phosphorylated Tau, neurofilament light chain, and glial fibrillary acidic protein as markers of convergent axonal injury and astrogliosis, along with reduced Aβ42 in both HAND and AD-related mild cognitive impairment (Clifford et al., 2009; Emrani et al., 2020). Corresponding plasma biomarkers mirror these CSF alterations, supporting the presence of a pathological continuum accessible to non-invasive screening (Chatterjee et al., 2023; Doecke et al., 2025). Tau-PET imaging with ^18^F-MK-6240 validates *in vivo* Braak-like progression, from early mesiotemporal involvement to neocortical spread, and correlates with CSF measures of Tau, p-Tau, and Aβ42 (Kreisl et al., 2022; Gérard et al., 2025). In HIV-patients virally suppressed over 60 years old, the increase of white matter hyperintensities is associated with bilateral parietal atrophy, while lower body mass index predicts greater frontoparietal/precentral atrophy, contributing to accelerated HAND-AD neuropathologic progression (Samboju et al., 2021). Prospective longitudinal studies report a 2–3-fold increased AD risk in older HIV positive people with prior HAND diagnosis, despite complete viral suppression with antiretroviral therapy, attributed to persistent chronic low-grade neuroinflammation (Mackiewicz et al., 2019). The APOEε4 allele worsens HAND cognitive severity and accelerates HAND-to-AD progression, correlating with greater Tau pathology accumulation and medial temporal lobe (hippocampal/entorhinal) atrophy (Emrani et al., 2020). Supplementary Tables 1, 2 briefly summarize the *in vitro* and *in vivo* studies reported in this review, respectively, and indicate the cell type or mouse model used, experimental approach, and major findings.

In conclusion, HAND-AD convergence necessitates hybrid biomarker/PET screening for early detection and trials targeting dual proteopathic–viral mechanisms (e.g., anti-Aβ/Tau plus anti-Tat strategies) in aging PLWH. This dual-hit model reframes HAND as a modifiable neurodegenerative risk factor with profound translational implications (Hategan et al., 2019; Mackiewicz et al., 2019).

## 9 Advantages and limitations of *in vivo* and *in vitro* models for tat and gp120-driven neurodegeneration

*In vivo* and *in vitro* experimental models for the study of Tat and gp120 involvement in neurodegeneration have been and will be fundamental to elucidate the molecular and cellular mechanisms activated by these viral proteins, leading to neurodegeneration and the pathogenesis of HAND. Due to the fact that neurons are not considered productively infected by HIV (Kovalevich and Langford, 2012b; Meyer et al., 2022), the use of both *in vivo* and *in vitro* systems is needed to obtain complementary insights into the direct and indirect pathogenic actions of Tat and gp120 in disrupting neuronal homeostasis through glial activation, neuroinflammation, and alteration of the neurovascuolar unit.

*In vitro* models have the advantage of facilitating the analysis of direct Tat and gp120 neurotoxic effects, including glial activation, oxidative stress, mitochondrial dysfunction, synaptic alteration, and neuroinflammatory signaling. In addition, *in vitro* models are widely used for genetic, and pharmacological manipulation thanks to their great reproducibility and low cost. On the other hand, experimental *in vitro* systems cannot fully reproduce cellular interactions, vascular components, immune responses, or the full anatomical organization of the CNS, even in organoids. And obviously, they fail to model chronic viral infection, BBB dynamics, and long-term progression of HAND in patients.

*In vivo* models are very useful for studying the physiological context in which multiple CNS cell types interact within a neurovascular and immune environment. These models are fundamental for elucidating the complex interactions between cells and between cells and the immune response, in experimental settings to establish the effects of prolonged exposure to viral proteins, and for evaluating BBB dysfunction and the role of chronic inflammation driven the disease progression. However, *in vivo* systems do not fully recapitulate human HIV neuropathogenesis, due to species-specific differences in immune responses, viral tropism, neuronal biology, and, more importantly, the inability of rodents to support productive HIV-1 infection. All these limitations prevent many findings from animal research from being translated into human applications. Finally, unlike in vitro models, transgenic models often rely on constitutive expression of individual viral proteins, without the possibility to modulate their levels, which may not accurately reflect their physiological levels or temporal regulation during natural infection.

Nevertheless, no available animal model fully recapitulates human HIV neuropathogenesis. Species-specific differences in immune responses, viral tropism, neuronal biology, and the inability of rodents to support productive HIV-1 infection limit the translational relevance of many findings. In addition, transgenic models often rely on constitutive or artificial expression of individual viral proteins, which may not accurately reflect their physiological levels or temporal regulation during natural infection.

Although each experimental model highlights only selected aspects of the disease, integrating mechanistic insights from cellular systems with the physiological complexity of animal models has substantially advanced our current understanding of HAND and continues to guide the development of novel neuroprotective and anti-inflammatory therapeutic strategies.

## 10 Conclusion

Extracellular HIV-1 Tat and gp120 proteins act as ongoing drivers of CNS neurodegeneration in people living with HIV, even when they are under effective cART. This happens through connected processes like mitochondrial dysfunction, synaptodendritic injury, chronic neuroinflammation, and similarities to Alzheimer’s disease-related conditions. These viral proteins, released from long-lasting CNS reservoirs, continue to cause neurotoxicity through receptor-mediated signaling, poor proteostasis, and self-reinforcing inflammatory cycles. This contributes to the high rates of HAND among aging individuals living with HIV. Targeting Tat and gp120 pathways using NLRP3 inhibitors, DRP1 modulators, or strategies that enhance clearance offers promising treatment options to reduce cognitive decline and the mixed HAND-AD symptoms. However, a significant obstacle common to all these adjunctive strategies remains CNS drug penetration: even compounds with strong in vitro efficacy may fail to reach therapeutic concentrations in the brain parenchyma due to BBB restrictiveness, and this limitation is not resolved by ART-induced viral suppression alone (Said and Venketaraman, 2025). Addressing this challenge, alongside a deeper mechanistic understanding of how Tat and gp120 sustain neurodegeneration within CNS reservoirs, will be essential to translate the therapeutic targets identified in this review into effective interventions for people living with HIV.
