# Supplementary material for: HIV-1 Tat and gp120 as key drivers of neurodegeneration in the central nervous system
Source: Front Microbiol. 2026 Aug 12;17:1895162. doi: 10.3389/fmicb.2026.1895162 (PMC13506959; doi:10.3389/fmicb.2026.1895162)
Supplement: Supplementary file 1 [file Table_1.docx]

| Study | Viral Protein | Cell Types Employed | Experimental Approach | Major Findings |
| --- | --- | --- | --- | --- |
| (Catani et al., 2000) | gp120 | Human neuroblastoma CHP100 cell line | Inhibition of CXCR4 and CCR5 receptors and analyses of cell death after exposure with gp120. | gp120 induces cell death in human neuroblastoma cells through the CXCR4 and CCR5 receptors. |
| (Fields et al., 2016) | gp120 | SH-SY5Y cells, differentiated in neuronal phenotypes  Primary rat cortical neurons | Cells were exposed to gp120 and mitochondrial alterations were assessed. | gp120 induces mitochondrial fusion, leading to neuroinflammation and neurodegeneration. |
| (Shah et al., 2016) | gp120 | SVGA cells  Primary cultures of human fetal astrocytes (HFA) | Assessment of ER stress in cells transfected with pSyngp120-JRFL or treated with different concentration of gp120; quantification of BiP and CHOP gene expression, evaluation of cell viability. | gp120 induces ER stress that could persist over a longer period and activates the intrinsic caspase pathway, leading to astrocyte death. |
| (He et al., 2020) | gp120 | Mouse primary microglia and primary cortical neurons were obtained from 10-day-old C57BL/6 mice  BV-2 cell line (mouse microglia) | Cells were expose to gp120 to assess inflammasome activation and pyroptosis induction; neurons were incubated with conditioned medium from gp120-treated microglia to study neurophaty and neuroinflammation induction. | gp120 induces neuroinflammation and neuropathy through NLRP3 inflammasome. |
| (Shrestha et al., 2023) | gp120 | SH-SY5Y cells | Treatment with gp120 and evaluation of its effect on glutamate excitotoxicity, ROS, and miR-499-5p. | gp120 acts by hyperactivating NMDAR leading to major influx of Ca2+ upregulating increased mitochondrial ROS production, and inducing mitochondrial shape changes. |
| (Agbey et al., 2025) | gp120 | Rat cortical neurons | Neurons were exposed to gp120 and morphology alterations were analyzed. | gp120 alters neuron ER morphology. |
| (Ensoli et al., 1990) | Tat | H9 cells  COS-1 cells | H9 cells were HIV-1-acutely infected and COS-1 cells were transfected with Tat plasmid. Effects of cell supernatants were studied. | Tat is released from HIV-1-acutely infected and from Tat-transfected cells; Tat-containing supernatants promote growth of AIDS-KS cells. |
| (Liu et al., 2000) | Tat | Rat pheochromocytoma PC12 neurons  Primary human neuron cultures. | PC12 cells were incubated with Tat; evaluation of Tat-lipoprotein receptor-related protein (LRP) binding; test Tat (different isoforms) neuronal uptake mediated by LRP  CAT assay in PC12 cells transfected with Tat. | Tat binding to LRP results in the efficient uptake of Tat into neurons and leads to inhibition of neuronal binding, uptake and degradation of physiological ligands for LRP. |
| (Avraham et al., 2004) | Tat | HBMEC cells | Cells were treated with Tat Abs or with the specific inhibitor SU-1498 to study BBB permeability changes induced by Tat. | Tat induction of permeability changes in HBMEC cells is time-dependent.  Tat impaires BBB permeability.  Tat activates FAK, leading to cytoskeletal reorganization and permeability changes. |
| (Eugenin et al., 2007) | Tat | Human fetal cortical tissue | Cells were transfected with Tat; study of the mechanism of neuronal apoptosis | Tat induce nitric oxide production leading to neuron and astrocyte apoptosis. |
| (Midde et al., 2012) | Tat | Rat striatal synaptosomes | Exposure of synaptosome with Tat; assessment of dopamine transporter (DAT) reactivity and dopaminergic tone. | Tat acts on protein kinase C by trafficking-dependent mechanism leading to inhibition of DAT function.  Tat alters the dopaminergic tone by regulating both DAT and vesicular monoamine transporter (VMAT2) proteins. |
| (Kim et al., 2013) | Tat | U-87 MG human glioma cells  SK-N-MC human blastoma  SH-SY5Y human blastoma  293FT human epithelial kidney cells | Assessment of interaction and co-localization between Tat and amyloid precursor protein. | Tat interacts with amyloid precursor protein and modify its processing, leading to increasing Aβ production. |
| (Chivero et al., 2017) | Tat | BV-2 microglial cell line  Primary rat microglia | Cells were exposed to Tat; assessment of inflammasome activation. | Tat can prime and activate the NLRP3 inflammasome in microglia leading to exacerbation of inflammation. |
| (Darbinian et al., 2020) | Tat | Primary human cortical neurons PBMC cells | Cells were incubated with conditioned media from HIV-1 infected PBMC, or Tat or Vpr viral proteins; assessment of mtDNA and nDNA damage, superoxide dismutase activity, ROS and ATP production. | Both conditions lead to higher mtDNA damage, than nDNA damage, leading to neurodegenerative processes  Tat is involved in the alteration of mitochondrial bioenergetics in human neurons.  Tat exerts toxic activity leading to apoptosis stimulation on mitochondrial survival. |
| (Hu et al., 2020) | Tat | Primary rat hippocampal neurons  Astrocytic cell line A172  HEK293 cells  Rat primary astrocytes isolated from whole brains of 1- to 3-day-old rats  Primary human astrocytes  Primary macaque astrocytes | Cells were exposed to Tat and were quantified miR-7 expression (intracellularly and in extracellular vesicles (EVs)) and the amount of excitatory and inhibitory synapses in presence of Tat. | Tat mediates the induction and release of EVs containing microRNA-7 that, after its uptake by neurons, leads to synaptic alterations. |
| (Kannan et al., 2022) | Tat | BV-2 cell line  Human primary microglia (HPM) cells, rat primary cortical and hippocampal neurons isolated from E18 rats | Characterization of EVs from media of BV-2 and HPM cells treated or not with Tat. | Exosomes produced cells activated by Tat, induce neuronal damage, dendritic and a synaptodendritic injury, and reduce excitatory neurotransmission. |
| (Yang et al., 2025) | Tat | Primary microglia and neuronal cells derived from 1-day-old C57BL/6 mice  BV-2  SH-SY5Y | Treatment with different concentration of Tat and were performed the assessment of IL-1β protein level after treatment, the observation of mitochondrial structure and the maesurement of ROS production. | Tat activates NLRP3 inflammasome and NF-κB signalling pathway leading to IL-1β secretion and neuronal damage. |
| (Teodorof-Diedrich and Spector, 2018) | Tat and gp120 | Human primary neurons | Neurons were incubated with gp120 and Tat and the translocation of autophagy and mithophagy markers were assessed. | gp120 and Tat alter mitophagy after induction of mitochondrial fragmentation. |

**Supplementary Table 1.** Overview of the studies included in this review, summarizing the *in vitro* models employed, the viral proteins investigated, the experimental approaches used, and the key findings reported in each study.
