# Supplementary material for: HIV-1 Tat and gp120 as key drivers of neurodegeneration in the central nervous system
Source: Front Microbiol. 2026 Aug 12;17:1895162. doi: 10.3389/fmicb.2026.1895162 (PMC13506959; doi:10.3389/fmicb.2026.1895162)
Supplement: Supplementary file 2 [file Table_2.docx]

| Study | Viral Protein | Animal Model | Experimental Approach and endpoint | Key Outcomes |
| --- | --- | --- | --- | --- |
| (Louboutin et al., 2010) | gp120 | Sprague–Dawley rats | A SV40-derived gene delivery vector, SV(gp120) was injected unilaterally and stereotaxically into the caudate-putamens at different doses; brain section were studied to assess inflammatory responses and microglial and astrocite cells proliferation due to gp120 expression in mice brains. | gp120 elicits proliferation of microglial cells, increases inflammatory response, induces astrocyte proliferation and  the gp120-induced neuroinflammation correlates with neuronal loss. |
| (Fields et al., 2016) | gp120 | gp120 tg mice (8 month old) | Endpoint: 6 weeks  Investigation of mitochondrial dynamics and mitochondrial morphology alterations. | gp120 induces mitochondrial fusion, leading to HIV-induced neuroinflammation and neurodegeneration. |
| (Shah et al., 2016) | gp120 | SJL/BL6/Sv129 mice expressing CCR5WT (3-4 month old) | Evaluation of ER stress markers in different region of mice brain, IRE1α, XBP-1s and pJNK expressions evaluation. | gp120 increases ER stress in prefrontal and parietal cortex with increase of the excitatory neurotransmitter leading to heightened damage to the dendritic cells in prefrontal cortex. |
| (Wenzel et al., 2019) | gp120 | gp120tg mice intercrossed with p75NTR null mice | Molecular, histological and behavioral analyses. | gp120 expression induces a decrease in spine density, can modify striatum spine morphology and induces abnormal levels of p75NTR leading to severe alteration of synapses. |
| (He et al., 2020) | gp120 | HIV-1 gp120 transgenic mice | Validation of neuroinflammation, neuronal survival, synaptic regeneration, cognitive performance using behavioral tests. | **gp120 neuroinflammation and neurodegeneration in HAND is correlated to NLRP3-driven pyroptosis in microglia.** |
| (Agbey et al., 2025) | gp120 | gp120 transgenic (tg) mice | Analysis of ER stress-associated proteins level and ER morphology. | gp120 alters neuron ER morphology. |
| (Giunta et al., 2009) | Tat | Inducible and brain-targeted HIV-1 Tat transgenic (GT-tg); Transgenic PSAPP (APPswe, PSEN1dE9) | Endpoints: 1 week and 3 months.  Mice GT-tg were induced to express Tat to simulate acute infection and mice PSAPP/Tat simulate long HIV infection. Bcl-xL expression was assessed in mouse brain sections or brain protein extracts and Tau phosphorylation was analysed in mice expressing Tat. | Tat increases amyloid plaques formation and induces, dose-dependently, neuronal damage and tau phosphorylation. |
| (Kim et al., 2013) | Tat | Amyloid precursor protein (APP)/presenilin-1 (PS1) transgenic mice | Stereotaxic injection of lentiviral vector for Tat were performed in hippocampi of 2-month-old APP/PS1 mice, followed by immunohistochemical assay. | Tat interacts with APP, modifying its processing, leading to increasing Aβ production. |
| (Fitting et al., 2013) | Tat | GFAP-driven, DOX-inducible, Tat transgenic male mice | Assessment of dendritic pathology and study of synaptic vesicle associated protein. Behavioural tests were conducted on mice. | Tat acts reducing dendritic spine density, altering cytological features of astroglia, and disrupting synaptic integrity and spatial learning memory. |
| (Leibrand et al., 2017) | Tat | Transgenic mice conditionally-expressing HIV-1 tat transgene (Tat+ mice) | Assessment of BBB permeability; evaluation of phagocytic macrophage/microglial-activity. | Tat destabilizes BBB integrity and upregulates activated, phagocytic, perivascular macrophages and microglia. |
| (Figarola-Centurión et al., 2023) | Tat | Sprague–Dawley rats | Bilateral stereotaxic injections of Tat were performed into the hippocampus and cells were isolated by immunoneuromagnetic techniques. Tert gene expression and protein quantification were conducted to study mitochondirla dysregulation and alteration of mitophagy. | Tat induces mitophagy and mitochondrial biogenesis dysregulation, leading to telomeric dysfunction. |

**Supplementary Table 2.** Overview of the studies included in this review, summarizing the *in vivo* models employed, the viral proteins investigated, the experimental approaches used and endpoint (if reported), and the key findings for each study.
